# Data on binding of L-tryptophan and bovine serum albumin by novel gold nanoparticles capped with amphiphilic sulfonatomethylated calixresorcinarenes

**DOI:** 10.1016/j.dib.2019.104241

**Published:** 2019-07-09

**Authors:** Ya V. Shalaeva, Ju E. Morozova, A.M. Shumatbaeva, I.R. Nizameev, M.K. Kadirov, I.S. Antipin

**Affiliations:** a Arbuzov Institute of Organic and Physical Chemistry, FRC Kazan Scientific Center of RAS, Arbuzov Str. 8, 420088 Kazan, Russia; b Kazan Federal University, Kremlevskaya St. 18, 420008 Kazan, Russia; c Kazan National Research Technical University Named After A.N. Tupolev - KAI, 10, K.Marx Str., Kazan, 420111, Russia

**Keywords:** Amphiphilic сalixresorcinarene, Macrocycle, Gold nanoparticle, L-tryptophan, Bovine serum albumin

## Abstract

The data provided in this paper are associated with the data in the «Binding of L-tryptophan and bovine serum albumin by novel gold nanoparticles capped with amphiphilic sulfonatomethylated calixresorcinarenes» paper (Shalaeva et al., 2019). Here, we represent i) the characterization data of calixresorcinarenes capped gold nanoparticles obtained by TEM and Vis- and IR spectroscopy; ii) the data giving the information about the interaction of modified AuNPs with L-tryptophan and bovine serum albumin by dynamic light scattering, spectrophotometry and fluorescence spectroscopy.

**Table:** Specifications table

Subject area	*Chemistry*
More specific subject area	*Nanoparticles, calixresorcinarenes, protein*
Type of data	*Tables, microscopy images, graphs*
How data was acquired	*Spectrophotometry: Perkin Elmer Instruments* *TEM: Libra 120* *IR: Bruker Vector 22* *Fluorimetry: Varian Cary Eclipse* *DLS: Zetasizer Nano instrument*
Data format	*Raw, analyzed, processed*
Experimental factors	*The nanoparticles were synthesized using calixresorcinarenes both as reducing and stabilizing agents in an aqueous solution at 25 °C in the air atmosphere with stiring during 2 hours.*
Experimental features	*Optical properties and sizes of nanoparticles, IR data, emission spectra of L-tryptophan and bovine serum albumin*
Data source location	*Arbuzov Institute of Organic and Physical Chemistry, FRC Kazan Scientific Center of RAS, Kazan, Russian Federation* *Kazan Federal University, Kazan, Russian Federation*
Data accessibility	*Data are available within this article*
**Related research article**	Ya.V. Shalaeva, Ju. E. Morozova, A. M. Shumatbaeva, I. R. Nizameev, M. K. Kadirov, I.S. Antipin, A. I. Konovalov, Binding of L-tryptophan and bovine serum albumin by novel gold nanoparticles capped with amphiphilic sulfonatomethylated calixresorcinarenes, J. Mol. Liq., 286 (2019) 110879. https://doi.org/10.1016/j.molliq.2019.110879

**Table:** 

**Value of the data** • The full study of multicomponent nanosized systems on the base of gold nanoparticles stabilized by supramolecular macrocycles, such as, sulfonatomethylated calixresorcinarenes, capable of multiple intermolecular interactions with a bovine serum albumin with the formation of «macrocycle-protein» complexes and the creation of cooperative supramolecular assembles with a protein in an aqueous solution is demonstrated. • Data obtained could help scientists who investigate the interactions of proteins with synthetic compounds to find out binding and recognition possibilities, any structural and functional changes in protein during the binding in particular under various external conditions and stability of proteins. • The TEM images, DLS, spectrophotometry and fluorimetry data presented here confirmed the formation of hybrid systems on the base of gold nanoparticles and amphiphilic sulfonatomethylated calixresorcinarenes with controlled size, aggregation, electro-optical and binding properties and could be useful for the understanding of the interaction mechanisms between protein and nanoparticles, give information about structural and functional changes of BSA and the nanoparticles. • This investigation has a value due to the promising possibility to use such systems for protein transport and visualization in biological media.

## 1 Data

**Fig. 1:**
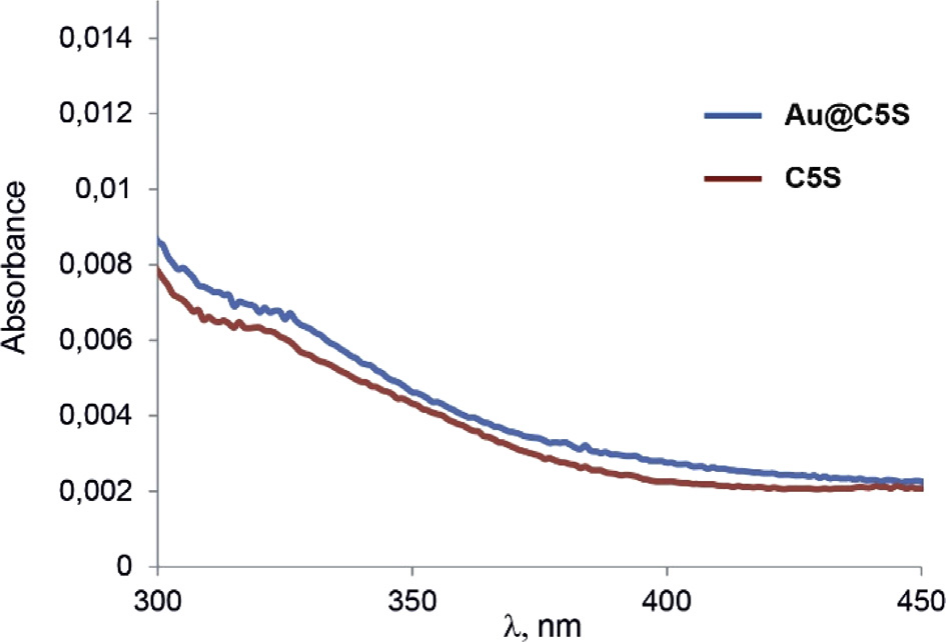
UV–Vis-spectra of aqueous solutions, containing **С5S** and Au@**С5S**, respectively (C = 10 μM).

**Fig. 2:**
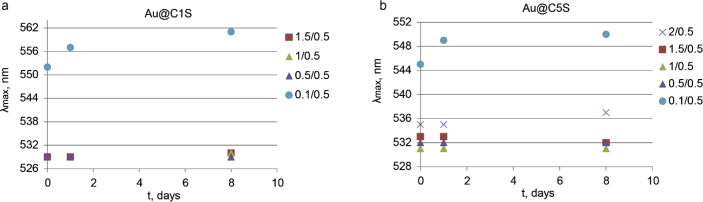
Time dependence of SPR maximum adsorption intensity of gold (*λ*_max_, nm) for Au@**С1S** (a) and Au@**С5S** (b).

**Fig. 3:**
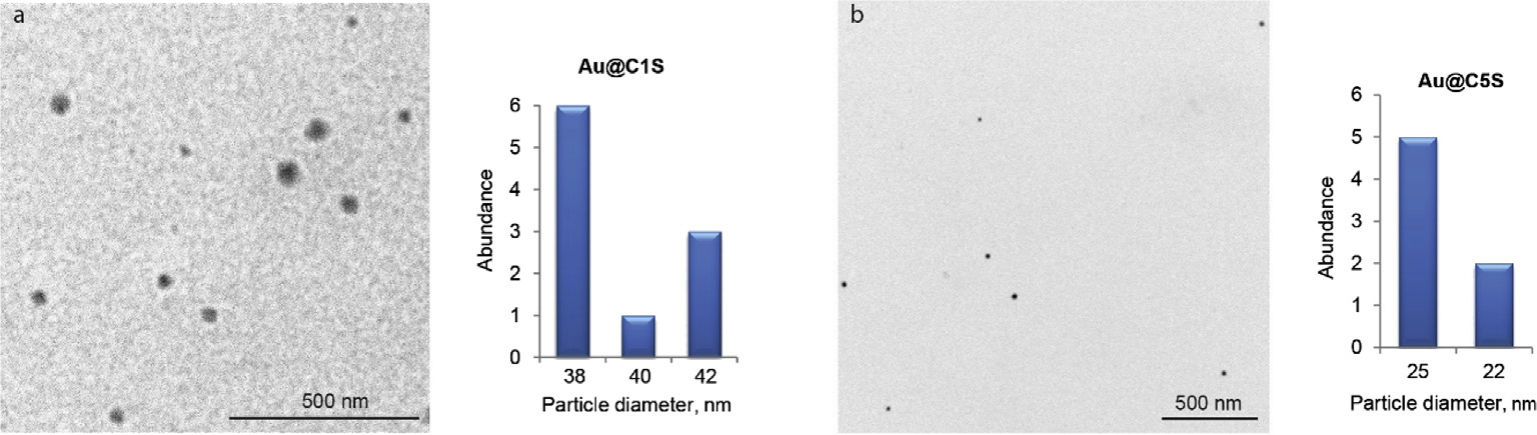
TEM images and histograms of size distribution of Au@**C1S** (а) and Au@**C5S** (b) nanoparticles (scale: 500 nm).

**Fig. 4:**
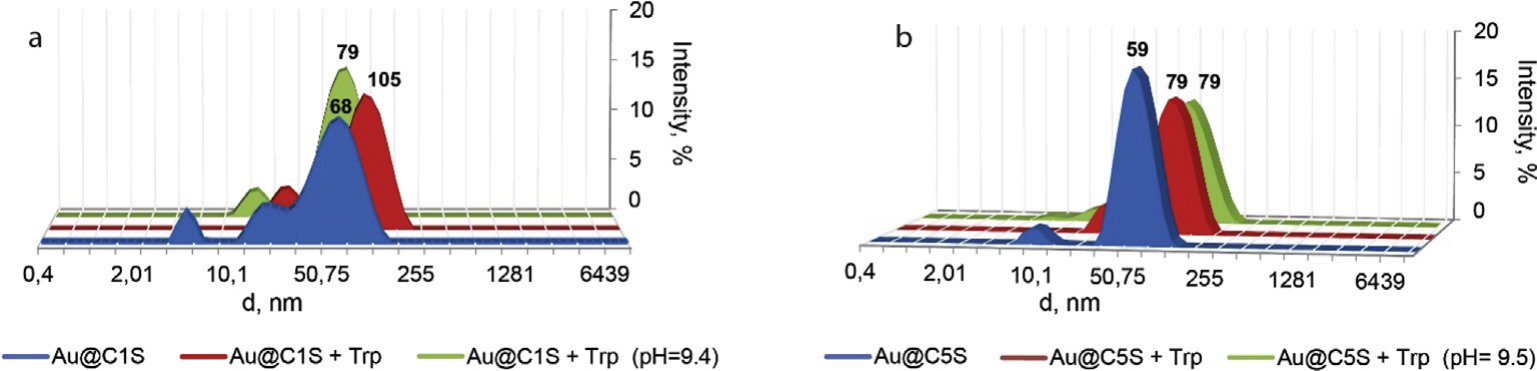
The intensity-averaged size distribution for aqueous solutions, containing (а) Au@**С1S**, Au@**С1S** + **Trp** (рН = 2.41) and Au@**С1S** +**Trp** (рН = 9.4) and (b) Au@**С5S**, Au@**С5S** + **Trp** (рН = 2.53) and Au@**С5S** +**Trp** (рН = 9.5) at 25 °C.

**Fig. 5:**
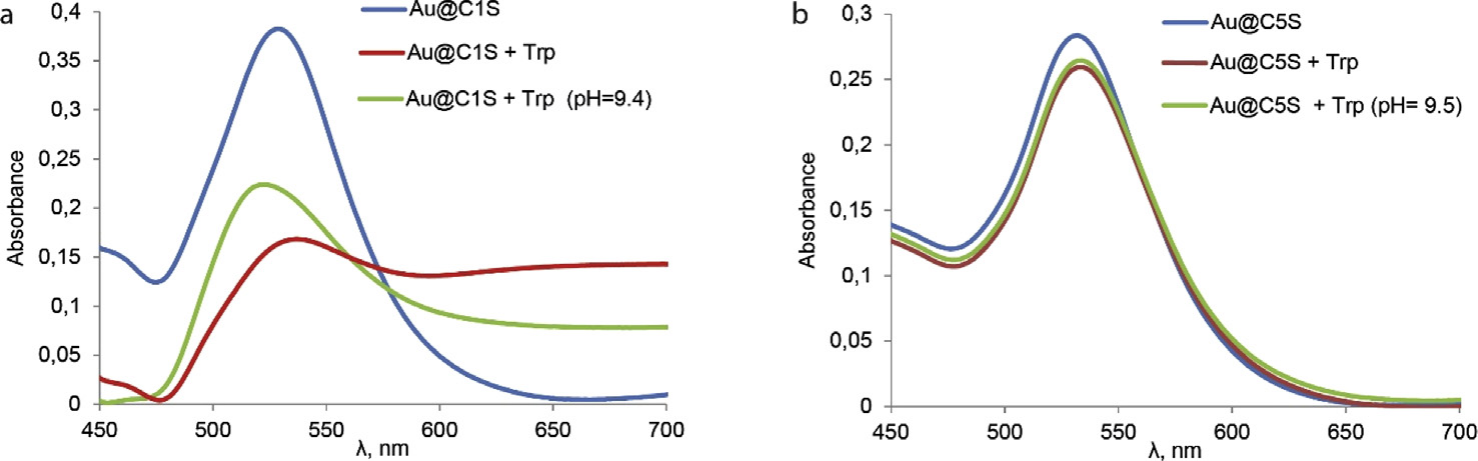
Vis-spectra of aqueous solutions, containing (а) Au@**С1S**, Au@**С1S** + **Trp** (рН = 2.41) and Au@**С1S** +**Trp** (рН = 9.4) and (b) Au@**С5S**, Au@**С5S** + **Trp** (рН = 2.53) and Au@**С5S** +**Trp** (рН = 9.5) at 25 °C (0.2 cm quartz path length cuvettes).

**Fig. 6:**
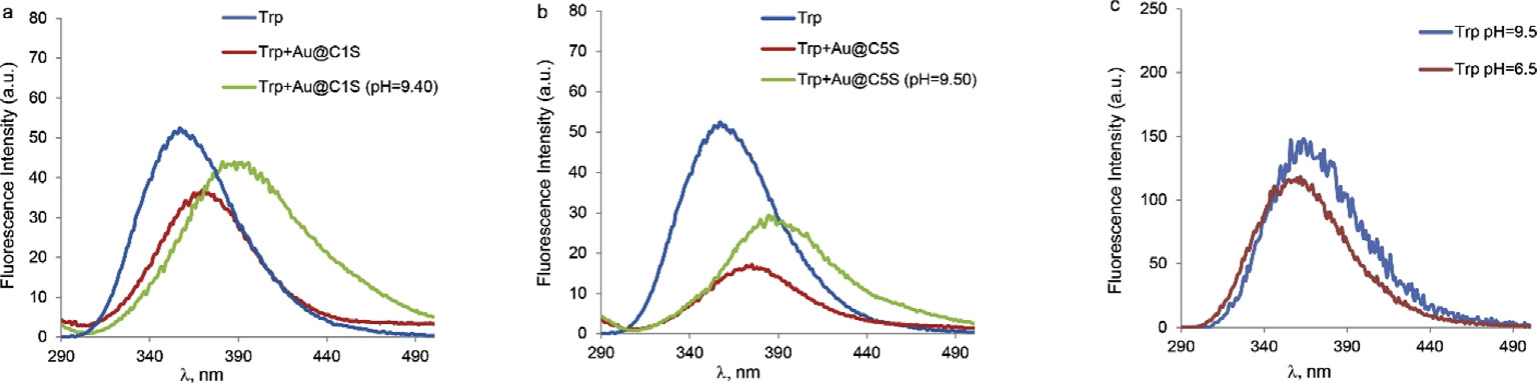
Fluorescence emission spectra of (a) **Trp** (0.1 mM), **Trp** in the presence of **С1S**@Au (0.5/0.5, mM) at spontaneous pH and at pH = 9.40; (b) **Trp** (0.1 mM), **Trp** in the presence of **С5S**@Au (0.5/0.5, mM) at spontaneous pH and at pH = 9.50; (c) **Trp** (0.1 mM) at spontaneous pH and at pH = 9.50; Ex. and Em. slits for **Trp** and **Trp** in the presence of nanoparticles have different values for clarity, V = 600 V.

**Fig. 7:**
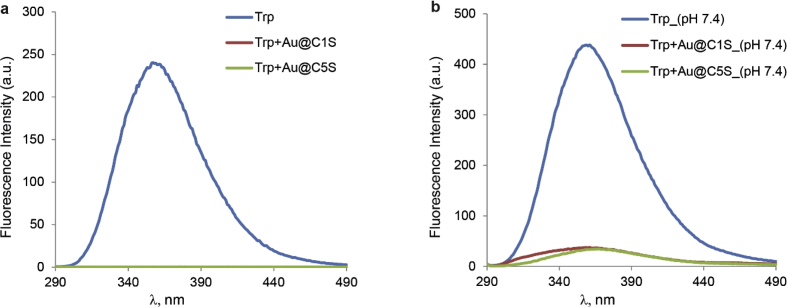
Fluorescence emission spectra of (a) **Trp** (0.1 mM), **Trp** in the presence of Au@**С1S** (0.5/0.5, mM) and Au@**С5S** (0.5/0.5, mM) at spontaneous pH (Ex. and Em. slits are 5 and 2.5 nm, respectively, V = 600 V); (b) **Trp** (0.01 mM), **Trp** in the presence of Au@**С1S** (0.1/0.1, mM) and Au@**С5S** (0.1/0.1, mM) in phosphate buffer, рН 7.4 (Ex. and Em. slits are 10 and 5 nm, respectively, V = 600 V).

**Fig. 8:**
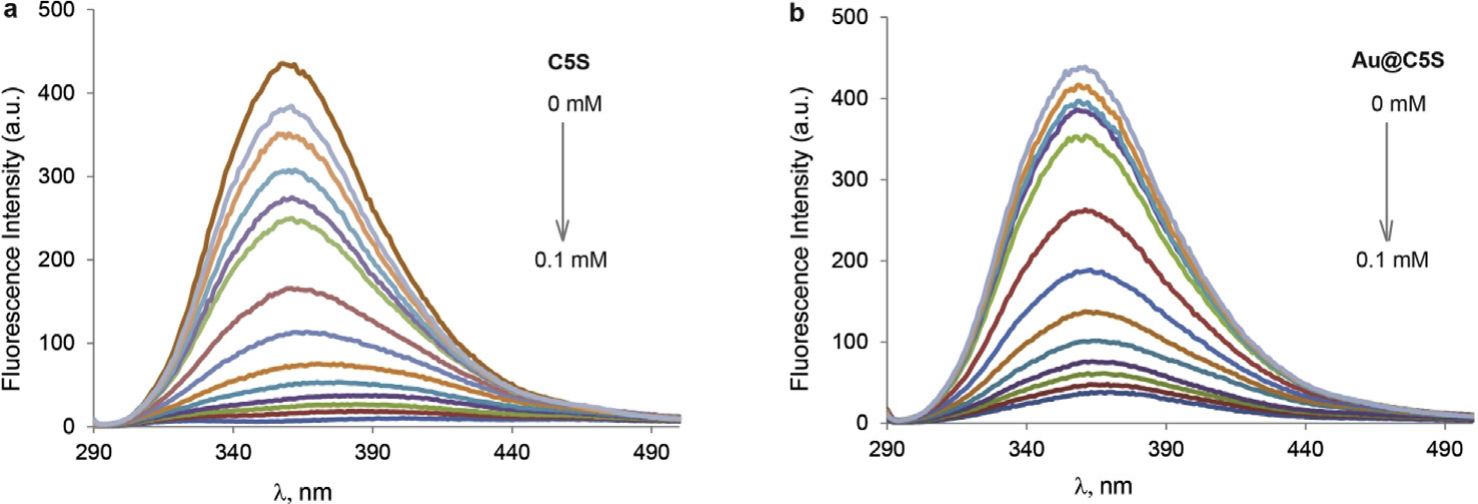
Fluorescence emission spectra of **Trp** (10 μM) in the absence and in the presence of **C5S** (from 0.001 to 0.1 mM) (a) and Au@**С5S** (from 0.001 to 0.1 mM) (b) (рН 7.4).

**Fig. 9:**
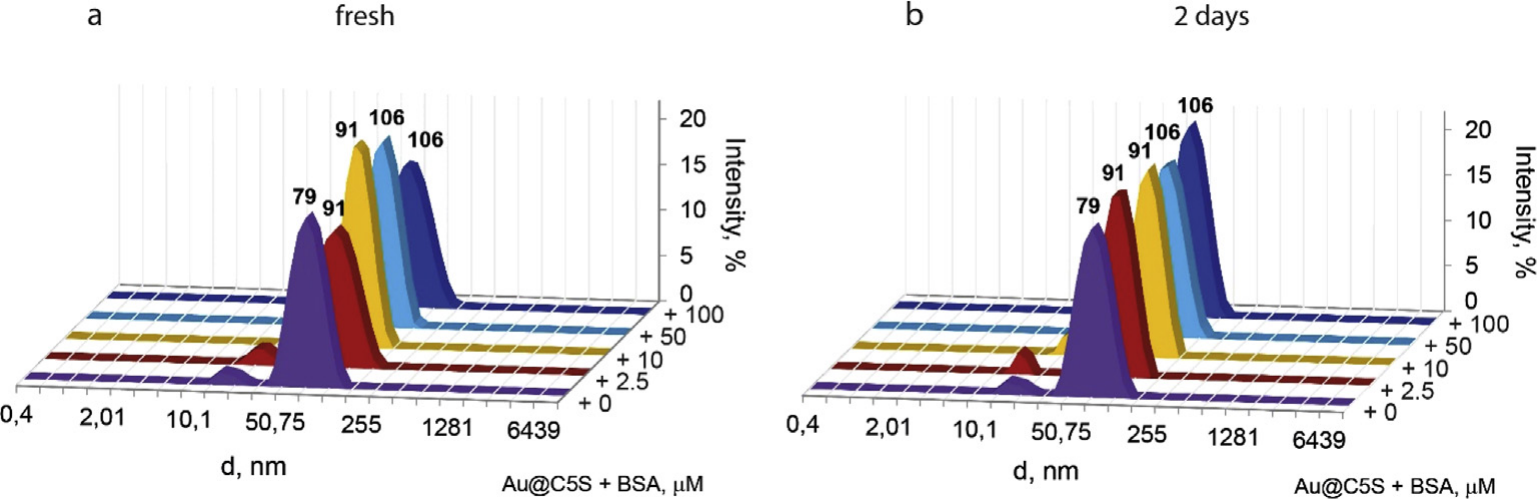
The intensity-averaged size distribution for aqueous solutions, containing Au@**С5S** (0.25/0.25, mM) in the presence of different amount of BSA (from 0 to 100 μM) at 25 °C: fresh (а) and in 2 days (b) (рН 7.4). For unbuffered soluions see [1].

**Fig. 10:**
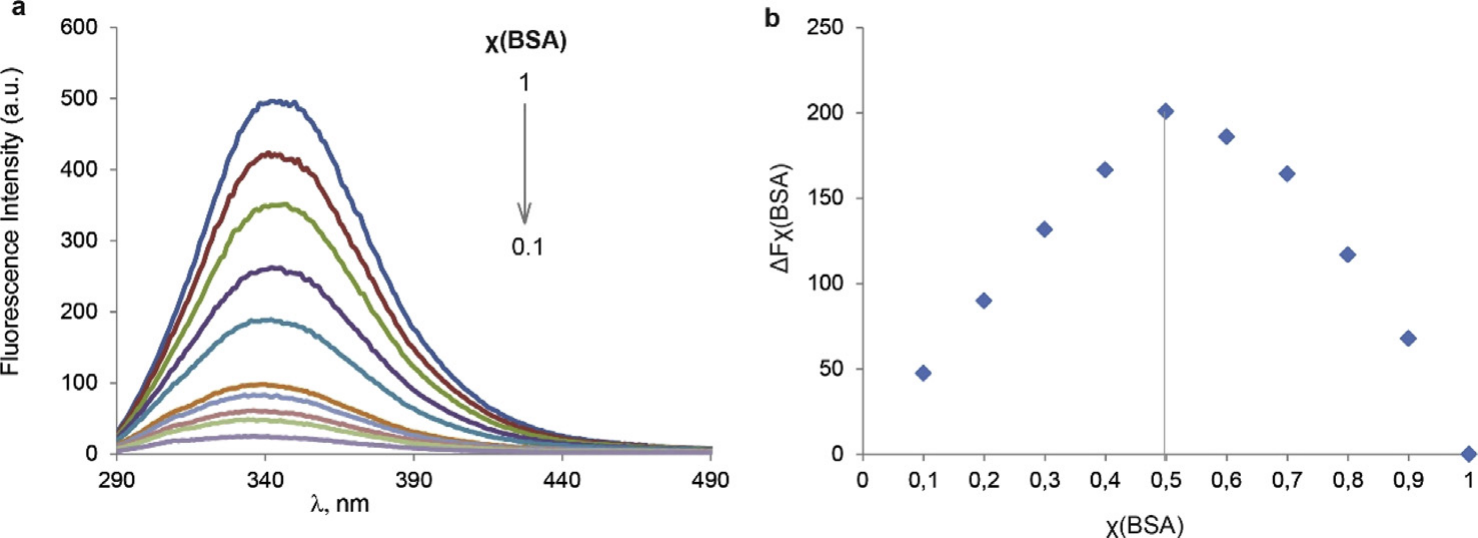
Fluorescence emission spectra for the Job's plot of BSA - Au@**С5S** systems (a) and Job's plot for the determination of stoichiometry of binding of BSA with Au@**С5S** (b), total concentration is 0.01 mM (рН 7.4).

This article contains the data about the synthesis of gold nanoparticles using sulfonatomethylated calixresorcinarenes with methyl (**C1S**) and pentyl (**C5S**) substituents on the lower rim both as reducing and stabilizing agents in an aqueous solution at different component ratio and structural characteristic (Table 2, IR data) of **C1S** and **C5S** macrocycles and obtained Au@**C1S** and Au@**C5S** nanoparticles. The values of averaged hydrodynamic diameters (*d, nm*) of Au@**C1S** and Au@**C5S** nanoparticles and their associates with **Trp** and BSA molecules are presented in Table 3, Table 4, Fig. 4 and Fig. 9. Fig. 3 illustrates the TEM images of Au@**C1S** and Au@**C5S** nanoparticles. There is also information about the spectral characteristic of nanoparticles and their changes during the interactions with **Trp** and BSA molecules (Table 1, Fig. 1 and Fig. 5, Fig. 6, Fig. 7, Fig. 8). Also, the data presented in this paper illustrate the stability of Au@**C1S** and Au@**C5S** nanoparticles during the time (Fig. 2). Fig. 10 demonstrates the stoichiometry of binding of BSA with Au@**С5S** (Job's plot).

**Table 1:** The values of the wavelength corresponding to the SPR maximum absorption intensity of gold nanoparticles (*λ*_max_, nm) modified by **C1S** and **C5S** macrocycles in an aqueous solution.

System	С_calix_/C_HAuCl4_, mM	*λ*_max_, nm
		fresh
	1.5/0.5	529
**C1S** + HAuCl_4_	1/0.5	529
	0.5./0.5	529
	0.1/0.5	552
**C5S** + HAuCl_4_	2/0.5	535
	1/0.5	533
	1.5/0.5	531
	0.5./0.5	532
	0.1/0.5	545

**Table 2:** IR data of **C1S***,* Au@**C1S***,***C5S** and Au@**C5S** nanoparticles (in KBr pallets, at macrocycle/HAuCl_4_ ratio of 0.5/0.5, mM).

System	ν_OH_, сm^−1^	ν_C_=_O,_ сm^−1^	ν_С_=_C,_ сm^−1^	ν_С-О_^-^_,_ сm^−1^	ν_as S_=_O_, сm^−1^	ν_s S_=_O_, сm^−1^
***C1S***	3438	–	1609	1476	1193	1044
*Au@****C1S***	3212	1718	1618	1476	1189	1042
***C5S***	3431	–	1610	1472	1193	1047
*Au@****C5S***	3423	1714	1627	1468	1209	1043

**Table 3:** The values of averaged hydrodynamic diameters of Au@**C1S**, «Au@**C1S +Trp**», Au@**C5S**, «Au@**C5S**+**Trp**» nanoparticles (*d, nm*) in an aqueous solutions, their intensities of scattering (*I, %*), polydispersity index (*PDI*) obtained by dynamic light scattering method.

System	C_calix_/C_HAuCl4_, mM	*d, nm* (*I, %)*	*PDI*
Au@**C1S**	0.1/0.5	91 (*22*)	*0.169*
Au@**C1S**	0.5/0.5	7 (*4*), 68 (*15*)	*0.431*
Au@**C1S** + **Trp**	0.5/0.5	105 (*13*)	*0.479*
Au@**C1S** + **Trp** (pH 9.4)	0.5/0.5	78 (*15*)	*0.415*
Au@**C5S**	0.1/0.5	79 (15)	*0.253*
Au@**C5S**	0.5/0.5	9 (*2*), 59 (*18*)	*0.350*
Au@**C5S** + **Trp**	0.5/0.5	79 (*14*)	*0.292*
Au@**C5S** + **Trp** (pH 9.5)	0.5/0.5	79 (*13*)	*0.415*

The error of the hydrodynamic particle size determination was <2%.

**Table 4:** The values of averaged hydrodynamic diameters of Au@**C5S**+BSA nanoparticles (*d, nm*) in an aqueous and PBS buffered solutions (pH = 7.4), their intensities of scattering (*I, %*), polydispersity index (*PDI*) obtained by dynamic light scattering method (fresh, after 2 and 7 days) and рH values of solutions.

System	рН	С_NPS_, mM	С_BSA_, μM	fresh		2 days		7 days	
				*d, nm* (*I, %)*	*PDI*	*d, nm* (*I, %)*	*PDI*	*d, nm* (*I, %*)	*PDI*
Au@**C5S** + BSA		0.5/0.5	0	9 (*2*), 59 (*18*)	*0.226*	9 (*2*), 59 (*18*)		9 (*2*), 59 (*18*)	
	5.59	0.5/0.5	2.5	68 (*22*)	*0.270*	91 (*21*)	*0.191*	91 (20)	*0.208*
	5.65	0.5/0.5	5	79 (*20*)	*0.183*	91 (*25*)	*0.177*	91 (19)	*0.231*
	6.26	0.5/0.5	10	91 (*18*)	*0.255*	91 (*21*)	*0.182*	91 (19)	*0.182*
	6.54	0.5/0.5	50	91 (*20*)	*0.164*	91 (*21*)	*0.174*	91 (21)	*0.181*
	6.76	0.5/0.5	100	91 (*18*)	*0.166*	91 (*18*)	*0.181*	91 (21)	*0.179*
Au@**C5S** + BSA	7.4	0.25/0.25	0	16 (*1*), 79 (*19*)	*0.228*	16 (*1*), 79 (*19*)		16 (*1*), 79 (*19*)	
	7.4	0.25/0.25	2.5	21 (*2*), 91 (*15*)	*0.182*	14 (*2*), 91 (*20*)	*0.206*	91 (22)	*0.193*
	7.4	0.25/0.25	10	91 (*22*)	*0.243*	91 (*21*)	*0.184*	91 (18)	*0.198*
	7.4	0.25/0.25	50	106 (*20*)	*0.197*	106 (*19*)	*0.190*	106 (19)	*0.208*
	7.4	0.25/0.25	100	106 (*15*)	*0.228*	106 (*21*)	*0.198*	106 (22)	*0.243*

The error of the hydrodynamic particle size determination was <2%.

^a^ – precipitation.

## 2 Experimental design, materials and methods

### 2.1 Materials

To synthesize the Au@**С1S** and Au@**С5S** nanoparticles we used the tetramethylensulfonated calixresorcinarenes with methyl (**C1S**) and pentyl (**C5S**) substitutes on the lower rim, which were obtained according to the previously reported procedure [2]. L-Tryptophan (**Trp**) and BSA from Sigma-Aldrich (Moscow, Russia) without any additional purification was obtained. HAuCl_4_·4H_2_O was kindly provided by Prof. E. Kh. Kazakova. All experiments were done in deionized water (3.5 μOm/cm).

### 2.2 Common synthetic procedure of Au@C1S and Au@C5S nanoparticles

To the aliquot of **CS** aqueous solution (C_CS_, mM; V_CS_, ml), deionized water (V, ml) and aliquot of HAuCl_4_ aqueous solution (C_Au_, mM; V_Au_, ml) were vigorously stirred at 25 °C. For 2 hours the reaction was complete finished that were confirmed by spectrophotometry method (Table 5). 2 ml of solution with final concentration of the components С_СS_/С_HAuCl4_, mM was obtained.

**Table 5:** Amounts of components during the synthesis of Au@**C1S** and Au@**C5S** nanoparticles.

Macrocycle	C_CS_, mM	V_CS_, ml	V, ml	C_Au_, mM	V_Au_, ml	С_СS_/С_HAuCl4_, mM
**C1S**	4	0.75	1.123	7.87	0.127	1.5/0.5
	4	0.5	1.373	7.87	0.127	1/0.5
	4	0.25	1.623	7.87	0.127	0.5./0.5
	4	0.05	1.823	7.87	0.127	0.1/0.5
**C5S**	4	1	0.873	7.87	0.127	2/0.5
	4	0.75	1.123	7.87	0.127	1/0.5
	4	0.5	1.373	7.87	0.127	1.5/0.5
	4	0.25	1.623	7.87	0.127	0.5./0.5
	4	0.05	1.823	7.87	0.127	0.1/0.5

### 2.3 Methods

***Uv–vis spectra*** were recorded on Lambda 35 UV–vis spectrometer (PerkinElmer Instruments, Shelton, CT, USA) in 0.2 cm quartz path length cuvettes with optical background correction. ***Transmission electron microscopy (TEM)*** images were obtained with Libra 120 (Carl Zeiss). The images were acquired at an accelerating voltage of 100 kV. Samples were dispersed on 300 mesh copper grids with continuous carbon-formvar support films. ***IR-spectra*** were recorded with Bruker Vector 22 FTIR Spectrometer (Bruker, Karlsruhe, Germany) with the wavelength range of 4000–400 cm^−1^. ***pH*** of aqueous solutions was measured at 25 °C with Thermo pH-meter (Thermo Electron, USA). ***Dynamic light scattering (DLS)*** measurements were сarried out on Zetasizer Nano instrument (Malvern Instruments, USA) with 10 mW 633 nm He–Ne laser light source and the light scattering angle of 173°. ***Emission spectra*** of L-tryptophan (**Trp**) and L-tryptophan-residues of BSA molecule were recorded on Varian Cary Eclipse spectrofluorometer (Agilent Technologies company production, USA) with the excited wave length at 279 nm using 1 cm path length quartz cuvettes at 25 °C.
